# COVID-19 and otorhinolaryngology: Returning to practice

**DOI:** 10.4102/sajid.v36i1.256

**Published:** 2021-03-26

**Authors:** Shivesh Maharaj

**Affiliations:** 1 Department of Neurosciences, Faculty of Health Sciences, University of the Witwatersrand, Johannesburg, South Africa

**Keywords:** COVID-19, SARS-CoV-2, coronavirus, otorhinolaryngology, ENT surgery, PPE, mitigation

## Abstract

This article aims to focus on key points and provide an overview of the current knowledge of the Coronavirus Disease (COVID-19); the increased susceptibility of otorhinolaryngologists to the virus; its effects and impact on the ENT practice; disruption of specialist clinic services; as well as associated risks in ENT surgical procedures. Mitigation strategies that can be employed to efficiently return to practice and ensuring the highest level of safety to both the patient and the otorhinolaryngologist is emphasised whilst simultaneously adapting to the new normal. Attention was given to understanding of the virus, its effect on the ENT discipline and practice, counter measures to mitigate and minimise risk to allow for continuation of ENT services once restrictions and lockdowns are progressively lifted. Otorhinolaryngological manifestations are common symptoms of COVID-19. Evidence suggests that the highest rates of nosocomial spread were seen amongst otorhinolaryngologists. The COVID-19 pandemic unexpectedly halted a majority of the otorhinolaryngology activities, which impacted service provision in the ENT practice. As the pandemic evolves, and with its duration unpredictable, this may necessitate a fundamental shift in the way otorhinolaryngology is practiced as there may be further global viral pandemics in future and the ENT fraternity has to now adapt to the new normal. Continued vigilance is imperative and strategies optimally implemented to ensure safe return to both ENT specialist clinic services and surgeries is vital. There are currently no uniform best-practice recommendations for otorhinolaryngology in the COVID-19 setting, although key strategies to prevent the virus spread have become evident to be able to effectively ‘flatten the curve’ of COVID-19 infections over time.

## Introduction

COVID-19 is caused by a new severe acute respiratory syndrome of coronavirus (SARS-CoV-2) discovered in Wuhan, China where the first cases where reported in December 2019. On 11 March 2020 the World Health Organization (WHO) declared the current COVID-19 outbreak a public health emergency of global concern and ultimately a pandemic.^1^ Given the magnitude of the coronavirus pandemic, its indeterminate pathogenesis and high rate of transmission, various widespread restrictions were imposed globally including government-mandated lockdowns in an effort to contain the viral spread.^2^ As on 05 August 2020 a total of 18.7 million confirmed COVID-19 cases globally, with 705 061 deaths and 11.9 million recoveries, were reported by WHO. In South Africa, for the same period, there were 521 318 confirmed cases, 8884 deaths and 363 751 recoveries reported.

With the rapid rise in COVID-19 cases, hospitals worldwide are reporting shortages of essential resources, namely intensive care unit (ICU) beds, medical equipment, testing materials and personal protective equipment (PPE), which is placing pressure on the healthcare system. Many institutions have postponed and/or cancelled elective and non-urgent procedures to conserve resources, limit exposure and minimise risk.^3^

According to Liu et al.,^4^ an increasing number of healthcare workers (HCWs) have been infected by the SARS-CoV-2 pandemic. They reported that data from China show more than 3300 HCWs were infected as of early March 2020, including at least 22 deaths, whilst reports from Italy demonstrate more than 5000 HCWs were infected. Healthcare workers in otorhinolaryngology-head and neck surgery in particular, often have close contact with the patients’ upper respiratory mucosa and because of the high viral load in the upper respiratory tract, are more vulnerable to SARS-CoV-2 related nosocomial infections.^4^ In another study conducted by Bann et al., it was stated that the first physician deaths during COVID-19 and severe acute respiratory syndrome (SARS) outbreaks were otorhinolaryngologists.^5^

In a study conducted by Topf et al., the authors mentioned that in light of the risk of nosocomial transmission of the SARS-CoV-2 to otorhinolaryngologists during aerosol generating procedures many organisations, namely, the American Head and Neck Society, the American Academy of Otorhinolaryngology Head and Neck Surgery, and the American Colleges of Surgeons are advocating for preoperative testing for all patients undergoing these high-risk procedures.^3^

The virus’s high rate of transmission prompted governments worldwide to implement various widespread restrictions in an effort to contain its spread and flatten the curve.^2^ Anecdotal reports indicated a changing ENT landscape initiated by the pandemic and subsequent lockdown; however, there has not been any documented evidence to confirm or refute this. Many centres throughout world initiated telemedicine consultations during lockdown periods, but this was not always possible in certain countries because of technology and infrastructure constraints.^6^

There have been reports of a decrease in the number of clinic attendees and surgical procedures in clinics throughout the world^7^, with many institutions cancelling or postponing elective and non-urgent procedures to free up inpatient beds, conserve resources and limit exposure of patients and HCWs.^7^

In order to resume ENT clinical activities and perform surgeries for urgent indications, it is critical that strategies are established and implemented to mitigate risk as it is not feasible to postpone emergency and/or urgent procedures until COVID-19 dissipates.^8^ Given the rapidly evolving situation, recommendations and approaches that are identified may be subjected to change as the pandemic continues to develop.

Personal protective equipment (PPE ) must be available and, depending on the associated risks like frequent aerosol generating procedures, use of high-speed drills, electro-cautery and suction irrigation, the correct PPE must be donned.^5^ In a study conducted by Liu et al.,^4^ it was emphasised that protection is not simply about PPE; it should encompass all principles of infection prevention and control, including disinfection immediately after surgery, as well as effective multidisciplinary collaborations with other specialist and the surgical team.^4^

The COVID-19 pandemic may be prolonged and returning to practice is essential. Therefore, it necessitates rethinking the way ENT services are provided and surgeries performed in order to protect the patient and all HCWs, in the current climate and beyond.

## Epidemiology, transmission and characteristics

SARS-CoV-2 is a pathogen that is phylogenetically similar to two previous zoonotic coronaviruses, namely, SARS-CoV and the Middle East Respiratory Syndrome coronavirus (MERS-CoV), which caused epidemics in 2002 and 2012.^9,10^

On 31 December 2019, a cluster of pneumonia cases of unknown cause was reported by health authorities in Wuhan, China. These cases predominantly had links to the Huanan Seafood Wholesale Market, which also sold live animals. Consequently, the virus is thought to have a zoonotic origin. The location of the virus’s origination is debated. The virus that caused the outbreak is SARS-CoV-2, a new virus that is closely related to bat coronaviruses, pangolin coronaviruses and SARS-CoV. Whilst it is suspected that the initial COVID-19 epidemic started through animal-to-human transmission, the current epidemic is being fuelled by human-to-human transmission and the virus has spread to more than 208 countries.^11^

Data from published epidemiological and virological studies highlight that viral transmission is primarily from symptomatic individuals to others who are in close contact through respiratory droplets, by being in direct contact with a person with COVID-19, or by direct contact with surfaces contaminated with SARS-CoV-2, as well as contact with own nasal or oral cavity or eyes.^11^

Van Doremalen et al. conducted an experimental study and reported that SARS-CoV-2 remained viable in aerosols for 3 h and survived up to 72 h on plastic and stainless steel, whilst on cardboard no viable virus was found after 24 h.^12^ Anecdotal reports of transmission of the infection from asymptomatic people are noted. Analysis of some scientific evidence has been published by the WHO on 29 March 2020, indicating the possibility of airborne transmission but this is not yet conclusive.^13^

The incubation time since exposure to SARS-Cov-2 is believed to reach 14 days. Majority of people develop COVID-19 after 5–6 days (range between 2 and 7 days) after being infected. The disease is contagious during this ‘pre-symptomatic’ period, thus the virus can be transferred to others before any clinical COVID-19 presentation.^14^

Coburn et al. reported that the reproductive number (*R*0) for the virus is approximately 2.2, meaning that on an average each person spreads the infection to two others. This is higher than that of SARS-CoV and MERS-CoV.^15^

## Clinical manifestations

There is a slight male predominance of 58.2% compared with females of 41.8%. Non-severe cases outweighed severe cases by a magnitude of 5, with higher age and any co-morbidity being associated with more severe disease.^14^ In a systematic review conducted by Krajewska et al., it was reported that most common presenting symptoms include fever (43% – 98%), dry cough (68% – 82%), fatigue (38% – 44%). Dyspnoea, sputum production and myalgia are reported in greater than 25% of the cases. Sore throat, rhinorrhoea, headaches, nausea and diarrhoea are less frequent and observed in mild to moderate cases. The authors also reported that South Korea, China and Italy presented that a significant number of individuals with COVID-19 was affected by hyposmia/anosmia and that COVID-19 could manifest as an isolated sudden anosmia, without any other symptoms. These patients should be tested and considered the source of the rapid spread of COVID-19.^16^

## Diagnosis

The specific molecular test presently recommended for SARS-CoV-2 detection is the real-time reverse transcriptase-polymerase chain reaction (RT-PCR) test. A positive RT-PCT test confirms the diagnosis of COVID-19 in vast majority of cases; however false-positive results can occur. Nasopharyngeal and oropharyngeal swabs are currently recommended for detecting and diagnosing the disease, with higher viral loads seen in the nasopharynx and are therefore, the preferred site to obtain a specimen. Broncho-alveolar lavage, endotracheal aspirates or sputum could be taken for testing.^16^ Wu et al. presented a case of COVID-19 with double negative tests obtained from nasopharyngeal swabs and suggested that inpatients with COVID-19 clinical symptoms, sputum or broncho-alveolar lavage fluid should be taken for examination. It was also speculated that saliva may serve as a potential, non-invasive material for diagnosing COVID-19.^17^ Authors from China reported that SARS-CoV-2 was detected in saliva specimens obtained from 91.7% of COVID-19 patients.^18^

Krajewska et al. reported that according to studies, computed tomography (CT) of the chest seemed to be beneficial and useful in diagnosing COVID-19 and is more sensitive than repeated RT-PCR tests. They noted that the sensitivity of RT-PCR tests and chest CT for diagnosing COVID-19 in suspected individuals reached 59% and 88%, respectively. Computed tomography of the thoracic cavity showing ground-glass opacities, infiltrates and broncho-vascular thickening consolidations strongly suggest SARS-CoV-2 infection. The authors, based on previous reports, speculated that during the pandemic, chest CT should be done in patients before ENT operations, which would be valuable in patients with negative PT-PCR.^16^

Common laboratory findings usually revealed normal or decreased levels of white blood cells, thrombocytes and lymphocytes; increased levels of erythrocyte sedimentation rate and C-reactive protein; normal levels of procalcitonin in a majority of the cases; increased levels of D-dimer, serum creatinine, creatinine phosphokinase; lactate dehydrogenase, prothrombin time and aminotransferase. High D-dimer concentrations and significant lymphopenia were related with high mortality. Patients with ENT manifestations of COVID-19 may present similar laboratory findings to patients at similar disease stage with other COVID-19 symptoms.^16^

## Management

Supportive care appropriate to the clinical severity is advised for the management of COVID-19 infections. Patients with severe disease are closely monitored and any signs of clinical deterioration (e.g. respiratory failure) are managed appropriately. Supportive treatment includes oxygen therapy in patients who experience shortness of breath. The target oxygen saturation (Sp0_2_) rates are ≥ 90% in non-pregnant adults and SpO_2_ ≥ 92% – 95% in pregnant patients. It was reported in CMS Council for Medical Schemes guideline in July 2020 that in the absence of an indication for endotracheal intubation, high-flow nasal oxygen, continuous positive airway pressure, or other non-invasive ventilation technique may be considered for adults with COVID-19 and acute hypoxaemic respiratory failure failing standard oxygen therapy.^19^

No vaccine against the SARS-CoV-2 is available yet. There is no known safe or effective therapeutic agent at present; however, several agents are currently being investigated in clinical trials. Various literature shows that Vitamin A, B, C, D, E, omega-3 polyunsaturated fatty acids, selenium, zinc and iron supplementation may be beneficial in COVID-19 management.^11^

Emphasis is placed in many studies relating to COVID-19 that standard infection prevention measures and disinfection must be applied stringently at all times.

## Otorhinolaryngology

During the early phase of the Wuhan outbreak, the highest rates of nosocomial spread were seen amongst otorhinolaryngologists as a result of the high viral load in the upper respiratory tract and the close contact otorhinolaryngologists have with the upper respiratory tract mucosa.^20,21^ Otorhinolaryngologists, especially ENT surgeons are also susceptible to COVID-19 transmission and are at high risk and could easily be infected by SARS-CoV-2, as they are involved in a variety of routine aerosol generating procedures that they may be required to perform, namely, endoscopic sinus and skull base surgery, abscess drainage, intubation, extubation, tracheostomy, maxillofacial trauma surgery, all of which represent a potentially high risk of viral aerosolization.^8^ It is not always feasible to postpone emergency and/or urgent procedures until COVID-19 dissipates.

It is recommended that otorhinolaryngologists be fully equipped when examining patients with unknown status. Patients with chronic ENT conditions and not requiring urgent consultation should be contacted telephonically. The number of staff attending the operating room theatre for ENT surgery should be limited to a minimum. For urgent ENT surgery or consultation, the correct PPE should be donned to ensure highest level of safety.

In a study conducted by Topf et al., the authors mentioned that in light of the risk of nosocomial transmission of the SARS-CoV-2 to otorhinolaryngologists during aerosol-generating procedures, many organisations, namely, the American Head and Neck Society, the American Academy of Otorhinolaryngology Head and Neck Surgery, and the American Colleges of Surgeons are advocating for preoperative testing of all patients undergoing these high-risk procedures.^3^

## COVID-19 mitigation recommendations for otorhinolaryngology practice and procedures

The COVID-19 pandemic may be prolonged and therefore necessitates rethinking what the new normal is, and the way clinical ENT is practiced in order to protect the patient and all HCWs, in the current climate and beyond. Therefore, implementing effective strategies to allow for surgeries or ENT consults for urgent indications and to prevent the SARS-CoV-2 infection amongst otorhinolaryngologists and ENT surgeons is of significance to the medical fraternity.

## Personal protective equipment

Personal protective equipment creates a barrier to protect the otorhinolaryngologists, other HCWs and the patient. Bann et al. defined ‘appropriate PPE’ as the standard-of-care procedure PPE specific for use with patients negative for COVID-19. ‘Enhanced PPE’ is defined as the use of N95 respirator and face shield or powered air-purifying respirator, disposable surgical cap, gown and gloves for patients with unknown, suspected, or positive COVID-19 status requiring invasive examination. The correct order of donning and doffing of PPE is vitally important.^5^

The Centers for Disease Control and Prevention (CDC) and the WHO recommend that attending HCWs wear a gown, gloves, goggles, visor and a medical mask.^22^

## In-office recommendations

An otorhinolaryngology group in Wuhan was able to avoid patient-to-physician infection using changes to clinic and operating room schedules, with scattered patient appointments rather than seeing patients en masse. The following guidelines may be used during nasal endoscopy.^23^

The patients’ nasal cavity and pharynx mucosa should be well anesthetized to reduce the cough and sneeze reflexes as a result of endoscopic manipulation. Gel-type topical anaesthetics rather than sprays should be used to minimise aerosol production. The smallest diameter endoscope is recommended as this has less of an irritant effect, thereby reducing the likelihood of coughing and sneezing.^24^

Nasal sprays should be avoided in patients with unknown, suspected, or COVID-19 positive status. Disposable cotton pledgets may be used for applying decongestants and topical anaesthesia.^5^

## Otolaryngologic procedures

As a result of high risk of viral aerosolization during ENT procedures, a plethora of cases have been postponed or cancelled. An improved understanding of procedures that generate aerosols and small droplets as well as the need to protect HCWs and conserve the limited supply of PPE is essential.^8^

Literature reveals that even though there are no formal guidelines or recommendations as yet on the best practices to reduce viral transmission during routine otolaryngological procedures in the COVID-19 setting, novel techniques have been reported recently to mitigate risks associated with oral intubation, extubation and endoscopic sinus surgery.^8^ These approaches should be considered by otorhinolaryngologists in order to allow safe continuation of surgery and outpatient clinic services. It must be emphasized that these methods are used as an adjunct to PPE usage and not as a substitute. Standard infection control precautions are standard practice and must be instituted at all times.^25^

If the patient is positive, surgery should be postponed as far as practically possible until they have recovered. If the patient requires emergency surgery, their COVID-19 status is unknown, or the patient is indeed infected with COVID-19, then surgery should be performed using mitigation strategies. All emergency cases and untested patients should be managed as COVID-19 positive until results are received, highlighting the need for the use of enhanced PPE.^5^ Clinical screening, early triage, isolation measures for high-risk individuals and adherence to PPE requirements is fundamental. Pre-operative COVID-19 testing of patients’ prior (at least 48–72 h) to surgery should be performed and patients should strictly quarantine until time of surgery.^5^

## Tracheostomy

Tracheostomy is one of the most frequent urgent ENT surgery performed. During the pandemic, every patient requiring emergency tracheostomy should be considered as COVID-19 positive. Those with an unknown or positive status, a cuffed and non-fenestrated tracheostomy tube should be used to prevent aerosolization.^16^ Video and disposable laryngoscopes should be used to maximise intubation success and minimise viral spread.^5^

### Role of tracheostomy

Indications for tracheostomy during the COVID-19 pandemic include emergent airway and prolonged mechanical ventilation. Patients requiring tracheostomy because of prolonged ventilation may have the procedure performed after 10–14 days of having tested positive for COVID-19, as this may reduce viral load and transmission during surgery.^5^

### Patients with existing tracheotomies infected with COVID-19

Tracheostomy patients should be connected to a closed ventilator circuit and standard droplet contact precautions are used, as would be for an endotracheally intubated patient on a ventilator. This closed system strategy was successfully used during the current outbreak in Hong Kong.^26^ The use of tracheostomy collars should be avoided whenever possible because of the aerosolization potential. Heat moisture exchanger (HME) is preferred.^5,16^

### Tracheostomy surgical procedure recommendations

Percutaneous tracheostomy may be a viable alternate for ICU patients; however, currently there are no clinical trials comparing the safety of the two procedures in an epidemic setting. Percutaneous tracheostomy could be performed as well as standard tracheostomy with minimal aerosolization spread, if the suggested precautions are strictly followed. Particular attention should be paid to the technical details aimed at reducing the time of exposure to aerosol.^27^

Mitigation techniques during tracheostomy procedure include preoxygenation, inserting the bulb of the endotracheal tube as close to the carina as possible when incising the trachea, thereby ensuring no puncture of the cuff of the endotracheal tube. During tracheostomy, the anaesthetist should be advised to render the patient apnoeic from the time the trachea is incised until the tracheostomy tube has been inserted and the cuff inflated. The use of electrocautery should be limited, and the procedure should be performed by the most senior member of the surgical team. Effective multidisciplinary collaboration between the otorhinolaryngologist and other specialists when performing procedures like tracheal intubation, non-invasive ventilation, tracheostomy in the COVID-19 positive patient is beneficial. The number of people in theatre should be kept to a minimum. If possible, the procedure should be performed in a negative pressure theatre with HEPA filtration.^5,16,27^

## Endoscopic sinus and skull base surgery

The viral load of SARS-CoV-2 is higher in the nasal cavity than in the throat, regardless of whether the patient is symptomatic or asymptomatic. Once aerosolized, SARS-CoV-2 particles may stay in the air for at least 3 h. During endoscopic sinus and skull base surgery, there are many mechanisms for possible aerosolization of mucus and virus particles, including powered instrumentation such as debriders and drills, suction electrocautery, as well as the use of saline irrigation either in the sinuses or to clean the endoscope. As any interaction of the airway mucus layer with high-speed flow causes aerosolization of mucus, even aerosolized spray of anaesthetic into the nasal cavity may cause aerosolization of mucus.^28^

In a study conducted by Chen et al., the authors noted that existing studies do suggest that the use of high-powered drills is associated with the generation of aerosols and small particles with the potential to transmit infectious diseases.^8^

## Endoscopic sinonasal procedures

Mitigation of transmission of COVID-19 may include the use of a nasal tent in endoscopic sinonasal surgery. This was demonstrated in a study conducted by Maharaj S, 2020. A clear, disposable isolation plastic sheet is used and draped as a tent over the operating area. This is connected to a suction to remove airborne particles and thus minimising transmission. It was described as an affordable (~4–6 US dollars) yet simple method to contain particle spread during high-risk aerosolization procedures during the COVID-19 pandemic era and beyond. Personal protective equipments must still be used by the surgeon and theatre staff. Limitations were identified by the author, together with learning curves, and it was emphasised that with proper training, planning and following the relevant protocols this method may be effectively used to mitigate risk and limit aerosol/droplet spread during these procedures.^29^

## Otologic surgical procedures including mastoidectomy

Mastoidectomy with a high-speed drill is regarded an aerosol generating procedure with the potential of high-risk viral transmission. Chen et al. described that the use of a low cost (~10 US dollars), simple barrier drape (1060 Steri-drape commonly used in ophthalmology procedures) that enclosed the surgical field (called an OtoTent) could be an effective mitigation strategy of aerosol and particle dispersion during a mastoidectomy in addition to appropriate use of PPE. Their study consisted of three test conditions, namely, open field microscope without OtoTent and microscope with OtoTent. The results indicated that the OtoTent quantitatively reduced particle dispersion beyond the boundaries of the drape. The authors acknowledged that various improvements and modifications can be made to the simple OtoTent design presented in their study.^8^

## ENT services during the COVID-19 pandemic: A South African experience

On 05 March 2020, the first known case of coronavirus was confirmed in South Africa. The subsequent initial increase in the number of cases resulted in government-mandated lockdown measures to slow the rate of spread and attempt to flatten the curve. This was initiated on 26 March 2020 and has resulted in the disruption of specialist clinic services in the ENT discipline.

Services provided by the Otorhinolaryngology, Head and Neck Surgery (ENT) department at a teaching hospital in Johannesburg, the Charlotte Maxeke Johannesburg Academic Hospital (CMJAH) were reviewed in a study conducted by Hari and Maharaj. It was noted that the unit continued to function as a quaternary referral centre with the specialist clinics and urgent/emergency surgery continuing during the pandemic (March 2020 to June 2020). The authors aimed to review patient presentations to their department and subsequent management over the first 85 days of lockdown, 26 March 2010 to 19 June 2010 (post-lockdown) and to compare this to the services rendered in the equivalent time period, 85 days (02 January 2020 to 25 March 2020) preceding lockdown (pre-lockdown). Data was extracted and results analysed.^30^

The results revealed that the functionality of their department changed markedly, especially during the initial phases of the lockdown, which was particularly stringent. There were 2160 visits scheduled in the pre-lockdown period, of which 1911 were completed (88.5%). During the equivalent post-lockdown time period there were 1220 scheduled visits and 937 patients attended (76.8%). The data comparisons between the pre- and post-lockdown periods showed significant decreases in both the number of patients booked (*p* = 0.01) and the attendance rates (*p* = 0.0001) at their specialist out-patient clinics. The greatest impact was seen in the general ENT (*p* = 0.009) and Head and Neck clinics (*p* = 0.017). A possible factor in the decrease in number of scheduled visits was the number of public holidays during the post-lockdown period, but even accounting for this the number of scheduled visits was meaningfully decreased.^30^

The authors reported that their out-patient clinic attendance rate post-lockdown decreased (76.8%) but paled in comparison to published data. Kasle et al. conducted a retrospective review within the Division of Otorhinolaryngology at the Yale School of Medicine to examine the quantitative changes in patient visits, modality of their care and subspecialty practice patterns during a selected period in the COVID-19 pandemic and compared it to the same period in 2019. Of the 5044 scheduled appointments, only 649 (12.9%) were completed in the 2020 period with the majority rescheduled or cancelled because of COVID-19. In addition, the majority (55.8%) of their completed visits were via tele-health, an impractical option in our setting.^2^ This discrepancy between the study conducted by Hari and Maharaj reported that their clinic non-attendance rates and that of Kasle et al. is consistent with suggestions of North American healthcare culture and possibly linked to their service being a government sponsored healthcare system.

It was noted during the analysis of the data that the monthly patient attendance at the outpatient clinics was fairly consistent in the pre-lockdown phase up to 25 March 2020 (31–35), in terms of patients seen per day. During the immediate period after lockdown, which included 4 clinic days in March and the following month of April, the patient attendance reduced drastically (10 per day). At this stage, South Africa was at level 5 lockdown, which entailed a severe curtailment of all activities. The months of May and June, saw a steady increase in the number of patients attending the outpatient clinics (15–21 per day), with the lockdown levels becoming more relaxed.^30^ The attendance numbers, however, remain lower than pre-lockdown levels. The rapid initial decline in patient visits mirrors that of Kasle et al. Their subsequent documented increase in completed visits was mainly attributed to tele-health visits, unfeasible in the outpatient clinics at CMJAH.^30^

In addition, Hari and Maharaj observed a decrease in attendance at all their clinics with the general ENT and head and neck clinics having the most significant declines in their attendance.^30^ This finding is consistent with that of Kasle et al. They observed that during the 2020 period, appointment completion rates dropped for all specialities and was highest for head and neck oncology (25.5%). This decline could be because of a fear of accessing the hospital, which is rightly considered a risky environment.^2^

With regards to theatre cases, the authors documented that all purely elective cases were postponed from 24 March 2020; however, oncology and emergency cases were still prioritised. This postponement led to a one-third decline in the ward admission rate during the post-lockdown period. The postponement is in line with measures adopted by comparable departments in Italy, a country which adopted a similar severe lockdown.^31,32^ The decrease in the number of surgical procedures performed during the post-lockdown period was deemed insignificant (*p* = 0.67). Ralli et al. in their study documented a 50.8% decrease in the number of ENT procedures done and this drastic reduction was explained by the timing of their study, which included the period during which Italy experienced their highest proportion of COVID-19 infections.^33^ Hari and Maharaj noted in their review that the post-lockdown period included only the initial phase of the very high numbers of infections documented to date in South Africa.^30^

Hari and Maharaj described variations in the proportion of the different procedures performed with the most significant changes seen in the numbers of tonsillectomies and sinus surgical procedures. This was readily explained by their departmental policy to cancel scheduled cases. Upper airway endoscopy consisting predominantly of direct laryngoscopy and surgical drainage of deep neck space infections remained the most common procedures performed in both periods. Both procedures were performed more frequently in the post-lockdown period. In the study by Ralli et al., a drastic reduction in the number of head and neck infections were noted.^33^ It is possible that data from the peak of South Africa’s pandemic may reflect similar changes.

Hari and Maharaj illustrated in their study, that despite a lockdown period there is still an ongoing need for specialist medical and surgical services and healthcare systems need to be tailored to manage all patients such that care is not shifted away from vulnerable groups and solely focused on COVID-19 patients. They concluded that unfortunately, clinic non-attendance and rescheduling of elective procedures in a system under constraint could have long-lasting repercussions on patient health outcomes which may be difficult to recover from timeously. A possible long-term solution would be the adoption of tele-health, a trend which would require significant commitment both, financially and in patient and healthcare education.^30^

## Conclusion

The COVID-19 pandemic has resulted in disruption of otorhinolaryngology and ENT services throughout the world. The head and neck region has one of the highest concentrations of virus particles and thus there has been a trend to postpone all elective surgery and reschedule out-patient clinics where possible. Even though there may be a reduction in the number of booked patients at outpatient clinics and elective procedures postponed or cancelled, there is still a need for the provision of urgent ENT services even in lockdown periods with increased numbers in certain areas such as head and neck endoscopy and tracheostomy.

In addition, this pandemic has led to the rapid adaptation of medical practice to incorporate strict adherence to infection prevention and control protocols. Otorhinolaryngology is a high-risk field as many of the common procedures are aerosol-generating in nature. This necessitates the judicious use of PPE and specific mitigation strategies in surgical practice to prevent the spread of infection, whilst providing much needed patient care.

As the COVID-19 pandemic evolves and with its duration unpredictable, this may necessitate a fundamental shift in the way otorhinolaryngology is practiced as there may be other global pandemics in future and the ENT fraternity has to now adapt to the new normal. Exploring effective mitigation strategies to be able to continue to perform procedures in a safe manner is vital for all.
